# Dietary patterns of children living in slums and their associated factors: a cross-sectional study, 2019-2022

**DOI:** 10.1590/S2237-96222024v33e20231275.en

**Published:** 2024-08-23

**Authors:** Gabriela Rossiter Stux Veiga, Bruna Merten Padilha, Lídia Bezerra Barbosa, Thaysa Barbosa Cavalcante Brandão, Telma Maria de Menezes Toledo Florêncio, Marilia de Carvalho Lima

**Affiliations:** 1 Universidade Federal de Alagoas, Faculdade de Nutrição, Maceió, AL, Brazil; 2 Universidade Federal de Pernambuco, Pós-Graduação de Saúde da Criança e do Adolescente, Recife, PE, Brazil

**Keywords:** Patrones Alimentarios, Consumo de Alimentos, Salud de los Niños, Vulnerabilidad Social, Estudios Transversales, Dietary Patterns, Food Consumption, Child Health, Social Vulnerability, Cross-sectional studies

## Abstract

**Objective:**

To identify dietary patterns and analyze factors associated with the consumption profile of socially vulnerable children, Maceió, state of Alagoas, Brazil, August 2019 to December 2021.

**Methods:**

This was a cross-sectional study; sociodemographic, anthropometric and food consumption variables were collected, factor analysis was used to identify dietary patterns; associations were analyzed using Poisson regression.

**Results:**

Among the 567 children studied, two dietary patterns were identified, healthy and unhealthy; age ≥ 24 months (PR = 2.75; 95%CI 1.83;4.14), male gender (PR = 0.66; 95%CI 0.49;0.87) and maternal schooling ≤ 9 years (PR = 0.61; 95%CI 0.46;0.81) was higher in the healthy pattern; the unhealthy pattern was associated with age ≥ 24 months (PR = 1.02; 95%CI 1.01;1.03) and male gender (PR = 1.46; 95%CI 1.08;1.98).

**Conclusion:**

The healthy pattern was more frequent in children aged ≥ 24 months, less frequent in male children and mothers with low level of schooling; children aged ≥ 24 months and males showed a higher prevalence of the unhealthy pattern.

## Study contributions

**Main results:**

Two dietary patterns were defined, healthy and “unhealthy”, which were associated with male gender, age ≥ 24 months and maternal schooling ≤ 9 years of study.

**Implications for services:**

The study data can assist health professionals dedicated to primary healthcare services, such as nutritionists, in planning interventions to promote healthy eating habits aimed at children.

**Perspectives:**

Prospective studies with these populations are necessary to assess the causality of the associations found in this study, aiming to plan more effective public health actions.

## RESUMEN

**Objetivo:**

Identificar hábitos alimentarios de niños socialmente vulnerables y analizar su relación con factores vinculados al perfil de consumo de Maceió, Alagoas, Brasil.

**Métodos:**

Estudio transversal de base poblacional llevado a cabo de agosto de 2019 a diciembre de 2021; se recogieron variables sociodemográficas, antropométricas y de consumo de alimentos, los patrones alimenticios se identificaron mediante análisis factorial; las asociaciones se analizaron mediante regresión de Poisson.

**Resultados:**

De los 567 niños estudiados se identificaron dos hábitos de alimentación, “saludable” y “no saludable”; edad ≥ 24 meses (RP = 2,75; IC_95_% 1,83;4,14), sexo masculino (RP = 0,66; IC_95_% 0,49;0,87) y educación materna ≤ 9 años (RP = 0,61; IC_95_% 0,46;0,81) se asociaron al patrón “saludable”; el patrón “no saludable” se asoció con la edad ≥ 24 meses (RP = 1,02; IC_95_% 1,01;1,03) y el sexo masculino (RP = 1,46; IC_95_% 1,08;1,98).

**Conclusión:**

El patrón “saludable” fue más frecuente en niños ≥ 24 meses y menos frecuente en niños varones y madres con menor educación; los niños de ≥ 24 meses y los varones tuvieron una mayor prevalencia del patrón “no saludable”.

## INTRODUCTION

Dietary patterns are defined as a set of foods frequently consumed, based on the usual diet, as people do not consume only isolated nutrients or foods.^1,2^ During childhood, in addition to behavioral factors, maternal characteristics such as age, schooling, quality of life, and others, as well as the implications of the social environment within which the family is situated, may be associated with dietary patterns, given that the mother and family play a fundamental role in child care.^3^

In developing countries, such as Brazil, social inequality is one of the determinants of food insecurity, defined as the situation when the general population, or a certain segment of it, lack access to adequate food.^4^ A significant portion of Brazilians live in social vulnerability, especially in municipalities where human development index (HDI) is lower. In the Northeast region, particularly in the state of Alagoas, the HDI is only 0.684 and 60.8% of households face difficulties in accessing food.^4^ The low purchasing power of families and low maternal schooling contribute to the sharing of unhealthy environments, reduced ability to purchase nutritionally adequate foods, and the provision of unhealthy items to children.^4,5^

Socially vulnerable people are those living in poverty and/or lack of access to basic rights to survival, such as food security.^6^ Despite the significance of this issue, studies covering socially vulnerable children are still scarce in the literature.^7,8^ Nevertheless, assessing child nutrition and its associated factors is crucial for the design of early interventions in the face of this challenge, when necessary, and for supporting public policies aimed at ensuring appropriate child development.

Habits formed in childhood are determinants for health in adulthood. Slum residents experience social vulnerability and food insecurity, with a higher likelihood of having an unhealthy diet and developing chronic non-communicable diseases in adulthood.^5^ Studying this group can contribute to reducing expenses on primary health care and providing higher quality service for the population as a whole.^7^

The objective of this study was to identify dietary patterns of socially vulnerable children and their associated factors.

## METHODS

This was a cross-sectional population-based study, conducted in the slums of Maceió, the capital of the state of Alagoas, in Northeastern Brazil, between August 2019 and December 2021, aiming to assess the sense of coherence among socially vulnerable mothers and its influence on the linear growth of their children.

Slums are comprised of populations lacking essential public services and, due to this condition, employ various strategies to autonomously and collectively meet their housing needs and associated uses, given the insufficiency and inadequacy of resources allocated to ensure citizens’ rights.^9^

The study included 10% of the 95 favelas identified in the 2010 Population Census (data available during the study period), selected randomly. All mothers living in these slums and who had at least one child aged between 6 months and 71 months and 29 days were considered eligible by the researchers. In order to minimize memory bias related to child information, when the mother had more than one child within the specified age group, the youngest child was selected. Pregnant women, mothers of preterm infants, or those with children having motor impairment, chronic diseases, or genetic syndromes interfering with growth and development were excluded from the study.

Data were collected during home visits by trained interviewers. The emergence of the COVID-19 pandemic interrupted data collection between March and November 2020.

Information on the child’s birth weight was obtained from the vaccination booklet. Additional information was collected through interviews with the mother. Maternal quality of life was assessed using the World Health Organization Quality of Life.^10^ Food security was assessed using the Brazilian Household Food Insecurity Measurement Scale (*Escala Brasileira de Insegurança Alimentar*),^11^ while data on the environmental sanitation were evaluated using questions from the Water, Sanitation and Hygiene protocol.^12^ Maternal sense of coherence (SOC)^13^ was scored between 13 and 65 points; scores above the median indicated strong SOC.

Data on the child’s dietary pattern were obtained using a form adapted from the National Demographic and Health Survey,^14^ gathering information on breastfeeding and the frequency of consumption of ultra-processed food groups, vegetables, fruits, candies and soft drinks, among others. The foods included in this instrument were fresh fruit juice, fruits, leafy greens, vegetables, processed meats, cookies/biscuits, processed juice, soft drinks, instant noodles, coffee, eggs, rice/noodles, beans and meat. Regarding breastfeeding history, mothers were asked if the child had been breastfed and for how long (in months).

The weight and length/height of the children and their mothers were measured using a Plenna portable digital scale (precision of 100 g and capacity of 150 kg) and mobile stadiometer manufactured by Alturexata (precision of 1 mm and capacity up to 2.13 m, which can be adapted for use as an infantometer), according to techniques standardized by Lohman.^15^

Anthropometric assessment was performed using the Anthro software (for children up to 5 years old) and Anthro Plus software (for children 5 years and older and adolescent mothers).The body mass index-for-age z-score was adopted for the analysis of nutritional status: underweight (< -2); normal weight (≥ -2 and ≤ +1); overweight (> +1 and ≤ +2) and obesity (> +2).^14^ mothers aged 19 years and older had their body mass index classified according to the World Health Organization (WHO) criteria.^16^ “Excess weight” was defined as the combination of the categories “overweight” and “obesity”. In order to measure the waist circumference of the mothers, an inelastic tape measure was used, and abdominal obesity was identified when waist circumference > 80 cm.^17^

The dependent variables were the two dietary patterns defined by the researchers, healthy and unhealthy, generated by principal component analysis (PCA), based on child dietary intake data reported by mothers when answering the food frequency questionnaire (FFQ). The dichotomization of the variables is described in the data processing item.

The independent variables were:

Socioeconomic and family information
household income per capita (in minimum wages, categorized by the median: ≥ ¼ and < ¼ of the minimum wage);
poverty level (according to the Alvarez score, categorized according to the score obtained: 45 to 54 points, higher low poverty; 20 to 44 points, lower poverty and extreme poverty);
number of people in the household (categorized by the median: ≤ 4 and > 4);
number of children in the household (categorized by median: 1 to 2; > 2);
type of drinking water (adequate; inadequate);
presence of sanitary sewage (adequate; inadequate);
waste management (adequate; inadequate);^12^12
food security (food security, 0 points; mild food (in)security, 1 to 5 points; moderate/severe food insecurity, 6 to 14 points).^11^

Maternal information
age (in years: 14 to 18; 19 to 29; ≥ 30);
schooling (in years of study: ≤ 9; >9).
Quality of life, analyzed according to the following parameters:
physical health (adequate; inadequate);
psychological aspects of behavior (appropriate; inappropriate);
social relationships (adequate; inadequate);
environment (adequate; inadequate);
SOC (strong; weak);
excess weight (yes; no);
abdominal obesity (yes; no);
height (≤ 150 cm; > 150 cm);
prenatal care (yes; no).

Child-related information
age (in months: < 24; ≥ 24);
sex (female; male);
birth weight (low weight, < 2,500 g; adequate weight, 2,500 to 3,999 g; and high weight, ≥ 4,000 g);
excess weight (yes; no);
breastfeeding history (yes; no); and
duration of breastfeeding (in months: ≥ 6; < 6).

Data were independently entered in duplicate, and analyzed using the Stata/SE software version 14.1 (StataCorp LP. College Station, TX, USA). Dietary patterns were defined based on food group consumption frequency data using the principal component analysis (PCA) statistical method, followed by Varimax orthogonal rotation. This method aims to reduce a large number of variables to a smaller number by grouping those that are strongly correlated, thus enabling the clustering of foods contained in the FFQ based on the degree of correlation between them. As a result of this statistical analysis, factor loadings were generated, and those with values ≥ 0.20 or ≤ -0.20 were considered.^18^ Items that did not show saturation were excluded from the correlation matrix because they did not meet the minimum value established for factor loading: 0.20 (exclusion of liver). Dietary patterns were defined after evaluating eigenvalues, with factors having eigenvalues > 1.5. The patterns were named according to the characteristics of the foods grouped in each factor.

The factor scores for each child were calculated. These scores were dichotomized (high consumption of food groups within dietary patterns: yes; no), considering high consumption of each dietary pattern when the consumption score was > 75th percentile (P75); and moderate/low consumption, when ≤ P75.19 Thus, a consumption score of a specific dietary pattern > P75 indicated greater adherence to the analyzed dietary pattern.

Descriptive analysis was performed, and the data were expressed as absolute and relative frequencies and respective 95% confidence intervals (95%CI).

The associations between dietary patterns [healthy and unhealthy (outcomes)] and the independent variables were assessed by calculating crude and adjusted prevalence ratios (PRs) and respective confidence intervals (95%CI), estimated using Poisson regression with robust variance adjustment. Analyses were performed separately for each dietary pattern. In the crude analysis, independent variables with a significance level of up to 20% (p < 0.2) were included in the adjusted analysis. The adjusted analysis was performed using the theoretical model proposed by Mendes et al.^20^ – with adaptations –, organized as follows: model 1 included family socioeconomic variables that showed p < 0.2 in the crude analysis; model 2 included maternal variables with p < 0.2 in the crude analysis, and the variables from model 1 with p < 0.05; and model 3, the final model, included child-related variables with p < 0.2 in the crude analysis and variables from models 1 and 2 with p < 0.05 (Supplementary material Figure 1). In each model, the backward stepwise technique was applied to eliminate variables that did not show a statistically significant association. Variables with p < 0.05 were considered significant in the final model.

**Figure 1:**
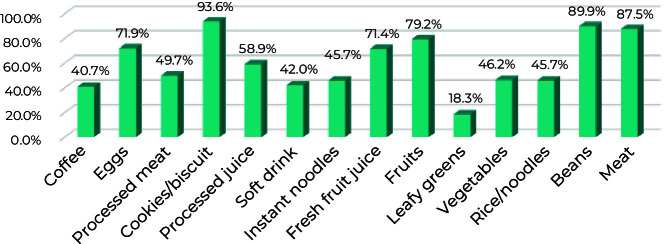
Consumption of foods that comprise the unhealthy and healthy dietary patterns of children living in slums (N = 567), Maceió, Alagoas state, Brazil, August 2019-December 2021

The research project was approved by the Research Ethics Committee of the Universidade Federal de Alagoas (CEP/UFAL): Certificate of Submission for Ethical Appraisal (CAAE) protocol No. 06340218.7.0000.5013. After being informed about the aspects of the research, the mothers signed the Free and Informed Consent Form opinion No. 3,375,586, approved on 06/06/2019.

## RESULTS

Data were collected from 602 eligible mother-child pairs, of which 35 pairs with outliers, noticed during data tabulation and considered losses of the study, were excluded. The final sample was comprised of 567 mother-child pairs. There was a slight predominance of male children (51.3%) and child’s age ≥ 24 months (57.1%). The average age of the mothers was 28.3 years (± 9.7 years), with most of them in the age group of 19 to 29 years (55.2%). There was a higher prevalence of overweight mothers (57.0%) with less than 9 years of schooling (60.3%), monthly household income per capita of less than ¼ of the minimum wage (67.9%) and moderate/severe food insecurity (61.4%) (Table 1).

**Table 1:** Characterization of the study sample according to socioeconomic, maternal and children living in slum variables (N = 567), Maceió, Alagoas state, Brazil, August 2019-December 2021

Variables	N	**%**	95%CI^a^
Family socioeconomic variables			
**Household income** *per capita* ^b^ **(** **minimum wage)**			
≥ 0,25	182	32.1	28.3;36.1
< 0,25	385	67.9	63.9;71.7
**Poverty level (Alvarez score points)**			
Higher low poverty (45 - 54)	159	28.0	24.4;31.9
Lower Poverty and Extreme Poverty (20 - 44)	408	72.0	68.1;75.6
**Number of people in the household**			
≤ 4	54	9.5	7.2;12.2
> 4	513	90.5	87.7;92.8
Number of children in the household			
1 - 2	447	78.8	75.2;82.1
> 2	120	21.2	17.9;24.8
**Drinking water**			
Adequate	175	30.9	27.1;34.8
Inadequate	392	69.1	65.2;72.9
**Sanitary sewage**			
Adequate	276	48,7	44,5;52,9
Inadequate	291	51,3	47,1;55,5
**Waste management**			
Adequate	194	34,2	30,3;38,3
Inadequate	373	65,8	61,7;69,7
**Food security**			
Mild security	219	38,6	34,6;42,8
Moderate/severe insecurity	348	61,4	57,2;65,4
**Maternal variable**			
**Age (years)**			
14-18	41	7,2	5,2; 9,7
19-29	313	55,2	51,0;59,3
≥ 30	213	37,6	33,6;41,7
**Schooling (years)**			
≤ 9	342	60,3	56,1;64,3
> 9	225	39,7	35,6;43,8
**Quality of life**			
**Physical health**			
Adequate	245	43,2	39,1;47,4
Inadequate	322	56,8	52,3;60,9
**Psychological aspects of behavior**			
Adequate	362	63,8	59,7;67,8
Inadequate	205	36,2	32,2;40,3
**Social relationships**			
Adequate	204	36,0	32,0;40,1
Inadequate	363	64,0	59,9;68,0
**Enviroment**			
Adequate	137	24,2	20,7;27,9
Inadequate	430	75,8	72,1;79,3
**Sense of coherence**			
Strong	235	41,4	37,4;45,6
Weak	332	58,6	54,4;62,6
**Maternal overweight**			
No	244	43,0	38,9;47,2
Yes	323	57,0	52,8;61,1
**Abdominal obesity**			
No	200	36,3	32,3;40,5
Yes	351	63,7	59,5;67,7
**Height (cm)**			
≤ 150	79	13,9	11,2;17,1
> 150	488	86,1	82,9;88,8
**Prenatal care**			
No	29	5,1	3,5;7,3
Yes	538	94,9	92,7;96,5
**Variables related to the child**			
Age (months)			
< 24	243	42,9	38,7;47,0
≥ 24	324	57,1	53,0;61,3
**Sex**			
Female	276	48,7	44,5;52,9
Male	291	51,3	47,1;55,5
**Birth weight**			
Adequate	445	85,4	82,1;88,3
Low weight	44	8,4	6,2;11,2
High weight	32	6,2	4,2;8,6
**Excess weight (child)**			
No	450	79,4	75,8;82,6
Yes	117	20,6	17,4;24,2
**Breastfeeding history**			
Yes	213	37,6	33,6;41,7
No	354	62,4	58,3;66,4
**Duration of breastfeeding (months)**			
≥ 6	336	59,3	55,1;63,3
< 6	231	40,7	36,7;44,9

a) 95%CI: 95% confidence interval; b) Cut-off point of 0.25 correspond to ¼ of the minimum wage.

The PCA identified two dietary patterns, unhealthy and healthy, which explained 38% of the total variance. The unhealthy eating pattern included coffee, eggs, processed meat, cookies/biscuits, processed juice, soft drinks and instant noodles; and the healthy dietary pattern consisted of fresh fruit juice, fruits, leafy greens, vegetables, rice/noodles, beans and meat (Table 2). Figure 1 shows the prevalence of consumption of the components of the unhealthy and healthy patterns.

**Table 2:** Factor loadings and dietary patterns identified in the food consumption of children living in slums (N = 567), Maceió, Alagoas state, Brazil, August 2019-December 2021

Food	**Dietary pattern**	
	“Unhealthy”	“Healthy”
Fresh fruit juice	-0.0409	0.3592
Coffee	0.2725	-0.0026
Fruits	-0.0028	0.3978
Leafy greens	-0.1087	0.3891
Vegetables	-0.1032	0.4333
Rice/noodles	0.0843	0.3000
Eggs	0.2840	0.1730
Beans	0.1519	0.2736
Meat	0.1099	0.3376
Processed meat	0.4093	-0.1125
Cookies/biscuit	0,3049	0.0987
Processed juice	0.4627	-0.0030
Soft drink	0.4126	-0.1268
Instant noodles	0.3453	0.0331
Variance	20.7	10.9

In the crude analysis, the unhealthy dietary pattern was associated with: (i) low level of maternal schooling, (ii) weak SOC, (iii) maternal overweight, (iv) abdominal obesity, (v) child’s age ≥ 24 months, (vi) male gender, and (vii) breastfeeding history. The healthy dietary pattern was associated with (i) low level of maternal schooling, (ii) male gender, (iii) child’s age ≥ 24 months, (iv) low birth weight, (v) childhood excess weight, and (vi) breastfeeding history; there were no socioeconomic variables associated with this pattern (Supplementary Table 1).

In the final adjusted analysis model, the unhealthy pattern was higher in children aged ≥ 24 months (PR = 1.02; 95%CI 1.01;1.03) and males (PR = 1.46; 95%CI 1.08;1.98). Regarding the healthy pattern, it was found that the frequency was higher in children aged ≥ 24 months (PR = 2.75; 95%CI 1.83;4.14) and lower in males (PR = 0.66; 95% CI 0.49;0.87) and when mothers had low level of schooling (PR = 0.61; 95%CI 0.46;0.81) (Table 3).

**Table 3:** Adjusted analysis between unhealthy and healthy dietary patterns of children living in slums (n = 567), related family and maternal socioeconomic variables, Maceió, Alagoas state, Brazil, August 2019-December 2021

**Variables**	**Unhealthy pattern**						**Healthy pattern**					
	**Model 1**		**Model 2**		**Model 3**		**Model 1**		**Model 2**		**Model 3**	
	**PR** ^a^ **(95%CI** ^b^ **)**	**p-value**	**PR** ^a^ **(95%CI** ^b^ **)**	**p-value**	**PR** ^a^ **(95%CI** ^b^ **)**	**p-value**	**PR** ^a^ **(95%CI** ^b^ **)**	**p-value**	**PR** ^a^ **(95%CI** ^b^ **)**	**p-value**	**PR** ^a^ **(95%CI** ^b^ **)**	**p-value**
**Family socioeconomic variables**												
**Household income** *per capita* ^c^ **(minimum wages)**												
≥ 0.25	1.00											
< 0.25	1.26 (0.88;1.79)	0.202										
**Poverty level (Alvarez score points)**												
Higher low poverty (45 - 54)							1.00					
Lower poverty and extreme poverty (20 - 44)							0.76 (0.56;1.01)	0.063				
**Presence of sanitary sewage**												
Adequate	1.00											
Inappropriate	1.16 (0.83;1.61)	0.380										
**Food security**												
Mild security	1.00											
Moderate/severe insecurity	1.20 (0.85;1.69)	0.305										
**Maternal variables**												
Age (years)												
14 -18									0.56 (0.24;1.29)	0.174		
19 - 29									1.00			
≥ 30									1.27 (0.95;1.69)	0.104		
**Schooling (years)**												
> 9	1.00		1.00						1.00		1.00	
≤ 9	1.39 (1.01;1.93)	0.047	1.28 (0.93;1.76)	0.124					0.62 (0.46;0.82)	0.001	0.61 (0.46;0.81)	< 0.001
**Quality of life**												
**Psychological aspects of behavior**												
Adequate			1.00						1.00			
Inadequate			1.08 (0.79;1.45)	0.676					0.82 (0.59;1.13)	0.218		
**Sense of coherence**												
Strong			1.00						1.00			
Weak			1.32 (0.96;1.82)	0.089					0.82 (0.65;1.16)	0.352		
**Overweight**												
No			1.00									
Yes			1.35 (0.85;2.14)	0.205								
**Abdominal obesity**												
No			1.00									
Yes			1.15 (0.70;1.87)	0.587								
**Child-related variables**												
**Age (months)**												
< 24					1.00						1.00	
≥ 24					1.02 (1.01;1.03)	< 0.001					2.75 (1.83;4.14)	< 0.001
**Sex**												
Female					1.00						1.00	
Male					1.46 (1.08;1.98)	0.015					0.66 (0.49;0.87)	0.004
**Birth weight**												
Adequate					1.00						1.00	
Low weight					1.17 (0.70;1.94)	0.553					1.34 (0.89;2.01)	0.158
High weigth					1.23 (0.69;2.19)	0.478					0.46 (0.18;1.21)	0.116
**Breastfeeding history**												
Yes					1.00						1.00	
No					1.19 (0.78;1.77)	0.402					1.03 (0.74;1.44)	0.852
**Excess weight**												
No											1.00	
Yes											0.70 (0.45;1.09)	0.112

a) PR: Prevalence ratio; b) 95% CI : 95% confidence interval of the relative frequency; c) Cut-off point of 0.25 correspond to ¼ of the minimum wage.

## DISCUSSION

In this study, two dietary patterns were identified: healthy and unhealthy. The highest consumption of the healthy dietary pattern was associated with child’s age ≥ 24 months, while the lowest consumption was associated with male children and mothers with lower level of schooling. These findings corroborate the hypothesis that lower level of schooling is associated with less healthy food choices.^21^ The lowest consumption of the unhealthy pattern was associated with the child’s age ≥ 24 months and male children.

The number of dietary patterns identified was similar to that found in a study analyzing the dietary pattern of Brazilian children.^22^ The number of patterns that can be identified in a given population varies depending on the diversity of food groups, sample size, and the pattern extraction techniques used in the studies.^3^ A systematic review aimed at identifying dietary patterns in children aged 7 to 10 years and their associated factors found a variable number of dietary patterns, from two to five, with a predominance of three.^3^

In studies conducted by Brazilian^23^ and American researchers,^24^ aimed at linking dietary patterns to metabolic syndrome and cardiovascular diseases, and identifying dietary patterns derived from a posteriori analysis, as decided in the present study, eggs were included in the unhealthy dietary patterns. The inclusion of high biological value protein source in this pattern was possibly due to the high social vulnerability of the analyzed population, which uses sausages and eggs as the primary protein sources in their meals because of their affordability. The high frequency of egg consumption among the children influenced the statistical analyses, resulting in eggs having a higher correlation with unhealthy foods.

Regardless of the number of dietary patterns obtained and component foods, it is crucial to identify factors associated with each pattern. A study conducted with children aged 13 to 35 months, in São Luís, the capital of the state of Maranhão, concluded that multiparity, lower level of maternal schooling and maternal age under 20 years were associated with lower consumption of healthy foods.^21^ Similar findings were found in a study involving 300 children aged 4 to 24 months in Porto Alegre, the capital of the state of Rio Grande do Sul, where lower level of maternal schooling was associated with a higher number of ultra-processed products consumed by children,^19^ corroborating our findings: lower maternal level of schooling was associated with a reduction in the healthy dietary pattern. A possible explanation for this finding is the fact that mothers with a higher level of education have greater access to information on healthy eating practices. Maternal schooling influences children’s lifestyle.^4^

Although no associations were found, it is widely acknowledged that environmental, nutritional, psychological, social and cultural factors may be related to eating behavior.^25^ Children rely on their parents/guardians to buy/prepare their meals, their eating habits are directly influenced by food beliefs and culture of their families.^25^ Among families that do not practice a diversified diet and show a low frequency of consumption of healthy foods, there is a higher likelihood of growth retardation in their children.^26^

One of the findings from the multiple analysis showed an association between the child’s age ≥ 24 months and a higher frequency of consumption of both healthy and unhealthy patterns, which was similar to that of a study conducted in the South of Brazil, where an association between dietary patterns of children aged 12 months and older was found;^27^ this finding was based on a greater independence of these children in choosing foods and their access to a wider variety of them, when compared to younger children^28^ – however, it is worth highlighting that the study population consisted of children from socially vulnerable families, predominantly without access to a diversified diet; as children grow older, they gain physiological capability and autonomy in food choices, within the possibilities existing in their environment.

The lack of association between male gender and higher frequency of consumption of the unhealthy dietary pattern among children is not a consensus in the literature. Studies have shown that male gender is associated with both healthy^29^ and unhealthy patterns;^1^ However, it can be hypothesized that, in socially vulnerable communities, boys have greater independence and autonomy, including in their food choices.

As for the healthy pattern, children of mothers with low level of schooling showed a lower frequency of consumption of foods in this pattern, a fact also observed in the cohort study conducted in São Luís, state of Maranhão.^21^ Low parental level of education may indicate a lack of adequate nutrition literacy, which promotes satisfactory self-care in matters related to children’s food and nutrition.^19^

According to a study conducted in Araraquara, state of São Paulo, in the period from 2015 to 2016, when evaluating families that were or were not Bolsa Família beneficiaries, those who were not covered by the Program were more likely to have a restricted dietary pattern, less likely to follow a healthy diet, regardless of the age of their members.^30^ A study conducted in the state of Paraíba also found that children with different types of social vulnerability were more likely to have an unhealthy dietary pattern.^5^

These data reinforce expectations: among children from socially vulnerable families, lower level of maternal schooling negatively impacts their dietary patterns. Adequate eating habits are extremely important in childhood because, over the long term, they can influence nutritional status and the development of chronic non-communicable diseases.^5,29^

The development of public policies aimed at promoting healthy eating for socially vulnerable children, especially those living in slums, presents a significant challenge for policymakers. Improving the diet of children living in disadvantaged environments is crucial for the development of these policies and can contribute to reducing unfavorable health outcomes, such as obesity.^4^

The study has limitations: the use of extensive questionnaires, the comprehension of which may be difficult for mothers with low level of maternal schooling to understand; and the interviewer bias, who is familiar with the population, may influence the way questions are asked, leading to biased responses. Both limitations imply information bias, although this was minimized by using questionnaires from instruments adopted for large national surveys and administered by trained researchers. Another limitation is the cross-sectional design, which prevents establishing causal relationships and may result in reverse causality, where the association between the variables differs from expectations. The use of multiple analysis mitigated this bias. Using a retrospective method (FFQ) to assess food consumption can lead to errors in the answers about food consumed, since it relies on the respondent’s memory. However, it is a method widely used in population-based surveys^3,23^ to assess habitual dietary intake of groups, and its limitation was mitigated by the short frequency adopted (the previous week). Another obstacle in the study methods, the temporal gap in data collection, did not compromise the homogeneity of the sample: individuals included in the study before the pandemic did not show statistically significant differences in socioeconomic and environmental conditions, when compared to those included after the pandemic outbreak (Supplementary Table 2).

A strong point of this research is the careful methodological approach in participant selection: all residents of the selected communities who met the eligibility criteria were recruited, minimizing the risk of selection bias. Another strong point was the use of validated instruments for data collection. These characteristics demonstrate the internal and external validity of the study; allowing the results to be extrapolated to similar populations in Brazil.

It can be concluded that the diet of socially vulnerable children was related to both intrinsic and extrinsic factors, the highest frequency of the healthy pattern was associated with age ≥ 24 months; and the lowest frequency of this pattern, with low level of maternal schooling and male children. Increased frequency of the unhealthy pattern was prevalent in children aged ≥ 24 months and in males. In order to elucidate the causality of variables associated with diet in this population, prospective studies are necessary.

## Supplementary Table 1

**Table:** Crude analysis between the unhealthy dietary pattern and the healthy dietary pattern of children living in slums (n = 567), related family and maternal socioeconomic variables, Maceió, Alagoas state, Brazil, August 2019-December 2021

Variables	Unhealthy pattern **(consumption > P75)**			Healthy pattern (consumption > P75)		
	**N (%)**	**Crude PR** ^a^ **(95%CI** ^b^ **)**	**p-value**	**N (%)**	**Crude PR** ^a^ **(95%CI** ^b^ **)**	**p-value**
**Famíly socioeconomic variables**						
**Household income** *per capita* ^c^ **(** **minimum wage)**						
= 0,25	36 (19.8)	1.00		44 (52.2)	1.00	
< 0,25	105 (27.3)	1.38 (0.99;1.93)	0.060	97 (25.2)	1.04 (0.76;1.42)	0.794
**Poverty level (Alvarez score points)**						
Higher low poverty (45 - 54)	36 (22.6)	1.00		48 (30.2)	1.00	
Lower poverty and extreme poverty (20 - 44)	105 (25.7)	1.14 (0.82;1.58)	0.449	93 (22.8)	0.76 (0.56;1.01)	0.063
**Sanitary sewage**						
Adequate	59 (21.4)	1.00		73 (26.5)	1.00	
Inadequate	82 (28.2)	1.32 (0.98;1.76)	0.063	68 (23.4)	0.8 (0.66;1.18)	0.397
**Food security**						
Mild security	45 (20.5)	1.00		60 (27.4)	1.00	
Moderate/severe insecurity	96 (27.6)	1.34 (0.98;1.83)	0.064	81 (23.3)	0.85(0.64;1.13)	0.267
**Maternal variables**						
**Age (years)**						
14 - 18	8 (19.5)	0.77 (0.40;1.48)	0.438	5 (12.2)	0.50 (0.21;1.17)	0.110
19 - 29	79 (25.2)	1.00		76 (24.3)	1.00	
= 30	54 (25.4)	1.00 (0.74;1.36)	0.977	60 (28.2)	1.16 (0.87;1.55)	0.316
**Schooling (years)**						
> 9	44 (19.6)	1.00		74 (32.9)	1.00	
= 9	97 (28.4)	1.45 (1.06;1.99)	0.02	67 (19.6)	0.60 (0.45;0.79)	<0.001
**Quality of life**						
**Psychological aspects of behavior**						
Adequate	83 (22.9)	1.00		98 (27.1)	1.00	
Inadequate	58 (28.3)	1.23 (0.92;165)	0.153	43 (21.0)	0.77(0.57;1.06)	0.113
**Sense of coherence**						
Strong	48 (20.4)	1.00		66 (28.1)	1.00	
Weak	93 (28.0)	1.37 (1.01;1.86)	0.043	75 (22.6)	0.80 (0.60;1.07)	0.135
**Excess weight**						
No	49 (20.1)	1.00		60 (24.6)	1.00	
Yes	92 (28.5)	1.42 (1.05;1.92)	0.024	81 (25.1)	1.02 (0.76;1.36)	0.894
**Abdominal obesity**						
No	37 (18.5)	1.00		51 (25.5)	1.00	
Yes	96 (27.3)	1.48 (1.05;2.07)	0.023	86 (24.5)	0.96(0.71;1.30)	0.794
**Child-related variables**						
Age (months)						
< 24	18 (7.4)	1.00		31 (12.8)	1.00	
= 24	123 (38.0)	5.12 (3.21;8.17)	0.000	110 (33.9)	2.66 (1.85;3.82)	<0.001
**Sex**						
Female	56 (20.3)	1.00		79 (28.6)	1.00	
Male	85 (29.2)	1.44 (1.07;1.93)	0.015	62 (21.3)	0.74 (0.56;0.99)	0.045
**Birth weight**						
Adequate	104 (23.4)	1.00		110 (24.7)	1.00	
Low weight	12 (27.3)	1.17 (0.70;1.93)	0.554	17 (38.6)	1.56 (1.04;2.35)	0.031
High weight	11 (34.4)	1.47 (0.89;2.44)	0.137	4 (12.5)	0.51 (0.20;1.28)	0.152
**Excess weight**						
No	117 (26)	1.00		121 (26.1)	1.00	
Yes	24 (20.5)	0.79 (0.53;1.16)	0.233	20 (17.1)	0.64(0.41;0.97)	0.038
**Breastfeeding history**						
Yes	38 (17.8)	1,00		42(19.7)	1.00	
No	103 (29.1)	1.63 (1.17;2.27)	0.004	99 (28.0)	1.42 (1.03;1.95)	0.032

a) PR: Prevalence ratio; b) 95%CI: 95% confidence interval of the relative frequency; c) Cut-off point of 0.25 correspond to ¼ of the minimum wage. a) Cutoff point of 0.25 correspond to ¼ of the minimum wage.

## Supplementary Table 2

**Table:** Socioeconomic and environmental characteristics of families in data collection during the pre-pandemic period and after the pandemic outbreak

Variables	Total		Pre-pandemic		After pandemic outbreak		p-value
	N = 567	%	N = 354	%	N = 213	%	
**Household income** *per capita* ^a^ **(** **minimum wage)**							
= 0.25	172	30.3	107	30.3	64	30.1	0.518
< 0.25	395	69.7	247	69.7	149	69.9	
**Poverty level (Alvarez score points)**							
Higher low poverty (45 - 54)	152	26.8	103	29.0	60	28.0	0.313
Lower poverty and extreme poverty (20- 44)	415	73.2	251	71.0	153	72.0	
**Type of drinking water**							
Adequate	174	30.7	110	31.0	65	30.5	0.893
Inadequate	393	69.3	244	69.0	148	69.5	
**Sanitary sewage**							
Adequate	183	32.3	110	31.1	66	31.0	0.985
Inadequate	384	67.7	244	68.9	147	69.0	
**Waste management**							
Adequate	193	34.0	121	34.2	71	33.5	0.644
Inappropriate	374	66.0	233	65.8	142	66.5	
**Food and nutrition security**							
Mild security	218	38.4	134	38.0	83	39.0	0.658
Moderate/severe insecurity	349	61.6	220	62.0	130	61.0	

a) Cutoff point of 0.25 correspond to ¼ of the minimum wage.
