# Supplementary material for: Dietary patterns of children living in slums and their associated factors: a cross-sectional study, 2019-2022
Source: Epidemiol Serv Saude. 2024 Aug 23;33:e20231275. doi: 10.1590/S2237-96222024v33e20231275.en (PMC11346682; doi:10.1590/S2237-96222024v33e20231275.en)
Supplement: Translation

# Translation: Padrões alimentares de crianças moradoras de favelas e fatores associados: um estudo transversal, 2019-2022

**DOI:** 10.1590/S2237-96222024v33e20231275.pt

**Authors:** Gabriela Rossiter Stux Veiga, Bruna Merten Padilha, Lídia Bezerra Barbosa, Thaysa Barbosa Cavalcante Brandão, Telma Maria de Menezes Toledo Florêncio, Marilia de Carvalho Lima

**Affiliations:** 1 Universidade Federal de Alagoas, Faculdade de Nutrição, Maceió, AL, Brasil; 2 Universidade Federal de Pernambuco, Pós-Graduação de Saúde da Criança e do Adolescente, Recife, PE, Brasil

**Keywords:** Padrões Alimentares, Consumo Alimentar, Saúde da Criança, Vulnerabilidade Social, Estudos Transversais

## Abstract

**Objetivo:**

Identificar padrões alimentares e analisar fatores associados ao perfil de consumo de crianças em vulnerabilidade social, Maceió, Alagoas, Brasil, agosto/2019-dezembro/2021.

**Métodos:**

Estudo transversal; coletaram-se variáveis sociodemográficas, antropométricas e de consumo alimentar, identificaram-se padrões alimentares por análise fatorial; analisaram-se associações mediante regressão de Poisson.

**Resultados:**

Das 567 crianças estudadas, identificaram-se dois padrões alimentares, saudável e não saudável; idade ≥ 24 meses (RP = 2,75; IC_95_% 1,83;4,14), sexo masculino (RP = 0,66; IC_95_% 0,49;0,87) e escolaridade materna ≤ 9 anos (RP = 0,61; IC_95_% 0,46;0,81) associaram-se ao padrão saudável; o padrão não saudável foi maior em idade ≥ 24 meses (RP = 1,02; IC_95_% 1,01;1,03) e sexo masculino (RP = 1,46; IC_95_% 1,08;1,98).

**Conclusão:**

Padrão saudável mais frequente em crianças ≥ 24 meses, menos frequente no sexo masculino e em mães de menor escolaridade; crianças ≥ 24 meses e do sexo masculino apresentaram maior prevalência do padrão não saudável.

## Contribuições do estudo

**Principais resultados:**

Foram definidos dois padrões alimentares, saudável e não saudável, aos quais se associaram o sexo masculino, a idade ≥ 24 meses e a escolaridade da mãe ≤ 9 anos de estudo.

**Implicações para os serviços:**

Os dados do estudo podem auxiliar profissionais de saúde dedicados aos serviços de atenção básica, como nutricionistas, no planejamento de intervenções para a promoção de hábitos alimentares infantis saudáveis.

**Perspectivas:**

Estudos prospectivos com essas populações são necessários para avaliar a causalidade das associações encontradas neste estudo, visando ao planejamento de ações de saúde pública mais efetivas.

## INTRODUÇÃO

Padrão alimentar é definido como um conjunto de alimentos frequentemente consumidos, com base na alimentação habitual, haja vista as pessoas não consumirem apenas nutrientes ou alimentos isolados.^1,2^ Durante a infância, além de fatores comportamentais, características maternas como idade, escolaridade, qualidade de vida, e outras, ademais das implicações do ambiente social em que a família se insere, podem estar associadas aos padrões alimentares, uma vez que a mãe e a família desempenham papel fundamental nos cuidados à criança.^3^

Países em desenvolvimento, como o Brasil, configuram a desigualdade social enquanto um dos fatores determinantes da insegurança alimentar, entendida como a ausência de acesso da população geral, ou de determinado segmento dela, a uma alimentação adequada.^4^ Grande parte dos brasileiros vive em vulnerabilidade social, principalmente nos municípios onde os índices de desenvolvimento humano (IDHs) são menores. No Nordeste do país, particularmente no estado de Alagoas, o IDH é de apenas 0,684 e 60,8% dos domicílios apresentam dificuldades de acesso à alimentação.^4^ O baixo poder aquisitivo das famílias e a baixa escolaridade materna contribuem para o compartilhamento de ambientes insalubres, menor poder de compra de alimentos com valor nutritivo adequado e oferta de itens não saudáveis às crianças.^4,5^

Pessoas em vulnerabilidade social apresentam condições de pobreza e/ou falta de acesso a direitos básicos para sua sobrevivência, como a segurança alimentar.^6^ Apesar da relevância do problema, estudos com crianças em vulnerabilidade social ainda são escassos na literatura.^7,8^ Todavia, a avaliação da alimentação infantil e seus fatores associados é importante para o delineamento de uma intervenção precoce diante desse desafio, quando necessária, e para o subsídio de políticas públicas que visem a um desenvolvimento infantil adequado. 

Hábitos formados na infância são determinantes para a saúde na vida adulta. Moradores de favelas vivem em vulnerabilidade social e insegurança alimentar, com maior probabilidade de terem uma alimentação não saudável e apresentarem doenças crônicas não transmissíveis na vida adulta.^5^ Estudar esse grupo pode contribuir para a diminuição dos gastos com a atenção primária à saúde e a oferta de um serviço de melhor qualidade, para a população como um todo.^7^

O objetivo deste estudo foi identificar padrões alimentares de crianças em vulnerabilidade social e seus fatores associados.

## MÉTODOS

Trata-se de um recorte de estudo transversal, conduzido em favelas de Maceió, capital do estado de Alagoas, Nordeste brasileiro, entre agosto de 2019 e dezembro de 2021, com o objetivo de avaliar o senso de coerência de mães em vulnerabilidade social e sua influência no crescimento linear dos filhos. 

Favelas são constituídas de populações carentes de serviços públicos essenciais, e, por conta dessa condição, utilizam diversas estratégias para atender, geralmente de forma autônoma e coletiva, às suas necessidades de moradia e usos associados, diante da insuficiência e inadequação de recursos destinados à garantia do direito cidadão.^9^

Foram incluídos no estudo 10% das 95 favelas identificadas no Censo Demográfico de 2010 (dados disponíveis no período do estudo), selecionadas aleatoriamente. Todas as mães residentes nessas favelas e que possuíssem pelo menos uma criança com idade entre 6 meses e 5 anos e 11 meses e 29 dias, foram elegíveis. Para minimizar o viés de memória relacionado às informações da criança, caso a mãe possuísse mais de um filho na faixa etária definida, selecionou-se o de menor idade. Foram excluídas do estudo gestantes, mães de filhos nascidos pré-termo ou portadores de deficiência motora, doenças crônicas ou síndromes genéticas que interferissem no crescimento e desenvolvimento. 

Os dados foram coletados em visitas domiciliares, por entrevistadores treinados. A emergência da pandemia de covid-19 interrompeu a coleta de dados entre março e novembro de 2020. 

Colheu-se a informação do peso da criança ao nascer, mediante consulta à caderneta de vacinação. As demais informações foram coletadas em entrevista com a mãe. Avaliou-se a qualidade de vida materna pelo World Health Organization Quality of Life.^10^ A segurança alimentar foi avaliada pela Escala Brasileira de Insegurança Alimentar,^11^ enquanto dados sobre o ambiente sanitário, por meio de questões previstas no protocolo Water, Sanitation and Health.^12^ O senso de coerência materno (SOC)^13^ foi pontuado entre 13 e 65 pontos; valores acima da mediana indicaram forte SOC. 

Dados sobre o padrão alimentar da criança foram obtidos utilizando-se um formulário adaptado da Pesquisa Nacional de Demografia e Saúde,^14^ reunindo informações sobre a amamentação e a frequência de consumo de grupos de alimentos industrializados, verduras, legumes, frutas, doces e refrigerantes, entre outros. Os alimentos constantes nesse instrumento foram suco natural, frutas, folhas, legumes, embutidos, biscoito/bolacha, suco industrializado, refrigerante, macarrão instantâneo, café, ovos, arroz/macarrão, feijão e carnes. Com relação ao histórico da amamentação, perguntou-se à mãe se a criança foi amamentada e por quanto tempo (em meses). 

Foram aferidos o peso e o comprimento/estatura das crianças e de suas mães, utilizando-se balança digital portátil Plenna (precisão de 100 g e capacidade de 150 kg) e estadiômetro móvel do fabricante Alturexata (precisão de 1 mm e capacidade até 2,13 m, podendo ser adaptado para infantômetro), conforme técnicas padronizadas por Lohman.^15^

A avaliação antropométrica utilizou-se dos *softwares* Anthro (para crianças até 5 anos) e Anthro Plus (para crianças acima de 5 anos e mães adolescentes). Adotou-se o escore Z do índice de massa corporal por idade para a análise do estado nutricional: baixo peso (< -2); peso normal (≥ -2 e ≤ +1); sobrepeso (> +1 e ≤ +2) e obesidade (> +2).^14^ Mães acima de 19 anos de idade tiveram seu índice de massa corporal classificado segundo o critério da Organização Mundial da Saúde (OMS).^16^ Denominou-se “excesso de peso” a junção das categorias “sobrepeso” e “obesidade”. Na aferição da circunferência da cintura das mães, usou-se fita métrica inelástica, identificando-se obesidade abdominal quando circunferência da cintura > 80 cm.^17^

As variáveis dependentes foram os dois padrões alimentares definidos pelos pesquisadores, saudável e não saudável, gerados pela análise fatorial por componentes principais, com base nos dados de consumo alimentar infantil informados pelas mães ao responderem ao questionário de frequência alimentar (QFA). A dicotomização das variáveis está descrita no item de processamento de dados.

As variáveis independentes foram:

Informações socioeconômicas e da família
renda familiar *per capita* (em salários mínimos, categorizada pela mediana: ≥ ¼ e < ¼ do salário mínimo);
nível de pobreza (pelo escore de Alvarez, categorizado segundo a pontuação obtida: 45 a 54 pontos, pobreza baixa superior; 20 a 44 pontos, pobreza baixa inferior e miséria);
número de pessoas no domicílio (categorizado pela mediana: ≤ 4 e > 4);
número de crianças no domicílio (categorizado pela mediana: 1 a 2; > 2);
tipo de água para consumo (adequada; inadequada);
presença de esgoto sanitário (adequado; inadequado);
manejo do lixo (adequado; inadequado);^12^
segurança alimentar (segurança alimentar, 0 ponto; (in)segurança alimentar leve, 1 a 5 pontos; insegurança moderada/grave, 6 a 14 pontos).^11^

Informações maternas
idade (em anos:14 a 18; 19 a 29; ≥ 30);
escolaridade (em anos de estudo: ≤ 9; >9).
Qualidade de vida, analisada de acordo com os seguintes parâmetros:
saúde física (adequada; inadequada);
comportamento psicológico (adequado; inadequado);
relações sociais (adequadas; inadequadas);
ambiente (adequado; inadequado);
SOC (forte; fraco);
excesso de peso (sim; não);
obesidade abdominal (sim; não);
altura (≤ 150 cm; > 150 cm);
acompanhamento pré-natal (sim; não);

Informações relacionadas à criança
idade (em meses: < 24; ≥ 24);
sexo (feminino; masculino);
peso ao nascer (baixo peso, < 2.500 g; peso adequado, 2.500 a 3.999 g; e peso elevado, ≥ 4.000 g);
excesso de peso (sim; não);
histórico de amamentação (sim; não); e
tempo de amamentação (em meses: ≥ 6; < 6).

Os dados foram digitados de forma independente, em dupla entrada, e analisados utilizando-se o *software* Stata/SE versão 14.1 (StataCorp LP. College Station, TX, EUA). A partir dos dados de frequência de consumo de grupos de alimentos, foram definidos os padrões alimentares pelo método estatístico de análise fatorial por componentes principais (ACP), seguido da rotação ortogonal Varimax. Esse método objetiva reduzir um grande número de variáveis a um número menor, agrupando aquelas fortemente correlacionadas, o que permite agrupar os alimentos contidos no QFA com base no grau de correlação entre eles. Como resultado dessa análise estatística, foram geradas cargas fatoriais, sendo consideradas aquelas com valores ≥ 0,20 ou ≤ -0,20.^18^ Foram excluídos da matriz de correlação os itens que não apresentaram saturação, por não atingirem o valor mínimo estabelecido para carga fatorial: 0,20 (exclusão do fígado). Os padrões alimentares foram definidos após avaliação de autovalores, com fatores de autovalor > 1,5. Foram nomeados os padrões formados de acordo com as características dos alimentos agrupados em cada fator. 

Foram calculados os escores de cada fator para cada uma das crianças. Esses escores foram categorizados dicotomicamente (consumo de grupos de alimentos dos padrões alimentares elevado: sim; não), considerando-se elevado o consumo de cada padrão alimentar quando o escore de consumo foi > percentil 75 (P75); e moderado/baixo, quando ≤ P75.^19^ Sendo assim, um escore de consumo de determinado padrão alimentar > P75 indicou maior adesão ao padrão alimentar analisado. 

A análise descritiva foi realizada, e os dados expressos em frequências absolutas e relativas e respectivos intervalos de confiança de 95% (IC_95_%).

As associações entre os padrões alimentares [“saudáveis” e “não saudáveis” (desfechos)] e as variáveis independentes foram verificadas por meio do cálculo das razões de prevalências (RPs) brutas e ajustadas e respectivos intervalos de confiança (IC_95_%), estimados pela regressão de Poisson com ajuste robusto de variância. As análises foram realizadas separadamente, para cada um dos padrões. Na análise bruta, as variáveis independentes ​​que apresentaram nível de significância até 20% (p < 0,2) foram incluídas na análise ajustada. A análise ajustada seguiu o modelo teórico proposto por Mendes et al.^20^ – com adaptações –, organizado da seguinte forma: o modelo 1 incluiu as variáveis socioeconômicas da família que, na análise bruta, apresentaram p < 0,2; o modelo 2 incluiu as variáveis maternas com p < 0,2 na análise bruta e as variáveis do modelo 1 que apresentaram p < 0,05; e o modelo 3, modelo final, incluiu as variáveis relacionadas à criança com p < 0,2 na análise bruta e as variáveis dos modelos 1 e 2 que apresentaram p < 0,05 (Figura Suplementar 1). Em cada modelo, aplicou-se a técnica *backward stepwise* para eliminação de variáveis que não apresentaram associação estatisticamente significativa. Variáveis com p < 0,05 foram consideradas significantes no modelo final.

O projeto da pesquisa foi aprovado pelo Comitê de Ética em Pesquisa da Universidade Federal de Alagoas: protocolo do Certificado de Apresentação para Apreciação Ética n^o^ 06340218.7.0000.5013. As mães, após serem informadas sobre os aspectos da pesquisa, assinaram o Termo de Consentimento Livre e Esclarecido nº parecer 3.375.586 e a data de aprovação foi 06/06/2019.

## RESULTADOS

Foram coletados dados de 602 pares de mães/filhos elegíveis, dos quais foram excluídos 35 pares com dados discrepantes, percebidos durante a tabulação de dados e considerados perdas do estudo. A amostra final foi de 567 pares de mães/filhos. Identificou-se discreto predomínio de crianças do sexo masculino (51,3%) e idade ≥ 24 meses (57,1%). A idade média das mães foi de 28,3 anos (± 9,7 anos), com maioria na faixa etária de 19 a 29 anos (55,2%). Houve maior prevalência de mães com excesso de peso (57,0%) e menos de 9 anos de estudo (60,3%), renda mensal *per capita* da família menor que ¼ do salário mínimo (67,9%) e famílias em insegurança alimentar moderada/grave (61,4%) (Tabela 1).

**Tabela 1:** Caracterização da amostra do estudo quanto a variáveis socioeconômicas, maternas e das crianças residentes em favelas (N = 567), Maceió, Alagoas, agosto de 2019-dezembro de 2021

Variáveis	N	%	IC_95_ **%** ^a^
Variáveis socioeconômicas da família			
**Renda familiar** *per capita* ^b^ **(salários mínimos)**			
≥ 0,25	182	32,1	28,3;36,1
< 0,25	385	67,9	63,9;71,7
**Nível de pobreza** **(escore de Alvarez pontos)**			
Superior (45 - 54)	159	28,0	24,4;31,9
Inferior e miséria (20 - 44)	408	72,0	68,1;75,6
**Número de pessoas no domicílio**			
≤ 4	54	9,5	7,2;12,2
> 4	513	90,5	87,7;92,8
**Número de crianças no domicílio**			
1 - 2	447	78,8	75,2;82,1
> 2	120	21,2	17,9;24,8
**Água de consumo**			
Adequada	175	30,9	27,1;34,8
Inadequada	392	69,1	65,2;72,9
**Esgoto sanitário**			
Adequado	276	48,7	44,5;52,9
Inadequado	291	51,3	47,1;55,5
**Manejo do lixo**			
Adequado	194	34,2	30,3;38,3
Inadequado	373	65,8	61,7;69,7
**Segurança alimentar**			
Segurança leve	219	38,6	34,6;42,8
Insegurança moderada/grave	348	61,4	57,2;65,4
**Variáveis maternas**			
**Idade (anos)**			
14 - 18	41	7,2	5,2; 9,7
19 - 29	313	55,2	51,0;59,3
≥ 30	213	37,6	33,6;41,7
**Escolaridade (anos)**			
≤ 9	342	60,3	56,1;64,3
> 9	225	39,7	35,6;43,8
**Qualidade de vida**			
Saúde física			
Adequada	245	43,2	39,1;47,4
Inadequada	322	56,8	52,3;60,9
**Comportamento psicológico**			
Adequado	362	63,8	59,7;67,8
Inadequado	205	36,2	32,2;40,3
**Relações sociais**			
Adequadas	204	36,0	32,0;40,1
Inadequadas	363	64,0	59,9;68,0
**Ambiente**			
Adequado	137	24,2	20,7;27,9
Inadequado	430	75,8	72,1;79,3
**Senso de coerência**			
Forte	235	41,4	37,4;45,6
Fraco	332	58,6	54,4;62,6
**Excesso de peso materno**			
Não	244	43,0	38,9;47,2
Sim	323	57,0	52,8;61,1
**Obesidade abdominal**			
Não	200	36,3	32,3;40,5
Sim	351	63,7	59,5;67,7
**Altura (cm)**			
≤ 150	79	13,9	11,2;17,1
> 150	488	86,1	82,9;88,8
**Acompanhamento pré-natal**			
Não	29	5,1	3,5;7,3
Sim	538	94,9	92,7;96,5
**Variáveis relacionadas à criança**			
**Idade (meses)**			
< 24	243	42,9	38,7;47,0
≥ 24	324	57,1	53,0;61,3
**Sexo**			
Feminino	276	48,7	44,5;52,9
Masculino	291	51,3	47,1;55,5
**Peso ao nascer**			
Adequado	445	85,4	82,1;88,3
Baixo peso	44	8,4	6,2;11,2
Elevado	32	6,2	4,2;8,6
**Excesso de peso (criança)**			
Não	450	79,4	75,8;82,6
Sim	117	20,6	17,4;24,2
**Histórico de amamentação**			
Sim	213	37,6	33,6;41,7
Não	354	62,4	58,3;66,4
**Tempo de amamentação (meses)**			
≥ 6	336	59,3	55,1;63,3
< 6	231	40,7	36,7;44,9

a) IC_95_%: Intervalo de confiança de 95%; b) Ponto de corte de 0,25 equivale a ¼ do salário mínimo.

A ACP identificou dois padrões alimentares, não saudável e saudável, os quais explicaram 38% da variância total. O padrão alimentar não saudável constituiu-se de café, ovos, embutidos, biscoito/bolacha, suco industrializado, refrigerante e macarrão instantâneo; e o padrão alimentar saudável, suco natural, frutas, folhas, legumes, arroz/macarrão, feijão e carnes (Tabela 2). A Figura 1 apresenta a prevalência do consumo dos elementos constitutivos dos padrões não saudável e saudável. 

**Figura 1:** Consumo de alimentos que compõem os padrões alimentares não saudável e saudável de crianças residentes em favelas (N = 567), Maceió, Alagoas, agosto de 2019-dezembro de 2021

**Tabela 2:** Cargas fatoriais e padrões alimentares identificados no consumo alimentar de crianças residentes em favelas (N = 567), Maceió, Alagoas, agosto de 2019-dezembro de 2021

Alimento	Padrão alimentar	
	**não saudável**	**saudável**
Suco natural	-0,0409	0,3592
Café	0,2725	-0,0026
Frutas	-0,0028	0,3978
Folhas	-0,1087	0,3891
Legumes	-0,1032	0,4333
Arroz/macarrão	0,0843	0,3000
Ovos	0,2840	0,1730
Feijão	0,1519	0.2736
Carnes	0,1099	0.3376
Embutidos	0,4093	-0,1125
Biscoito/bolacha	0,3049	0,0987
Suco industrializado	0,4627	-0,0030
Refrigerante	0,4126	-0,1268
Macarrão instantâneo	0,3453	0,0331
Variância	20,7	10,9

Na análise bruta, associaram-se ao padrão alimentar não saudável a baixa escolaridade materna, SOC fraco, excesso de peso materno, obesidade abdominal, idade da criança ≥ 24 meses, sexo masculino da criança e histórico de amamentação. Ao padrão saudável, mostraram-se associados baixa escolaridade materna, sexo masculino da criança, idade da criança ≥ 24 meses, baixo peso ao nascer, excesso de peso infantil e histórico de amamentação; nenhuma variável socioeconômica esteve associada a esse padrão (Tabela Suplementar 1)

No modelo final da análise ajustada (Tabela 3), o padrão não saudável esteve mais presente em crianças com idade ≥ 24 meses (RP = 1,02; IC95% 1,01;1,03) e do sexo masculino (RP = 1,46; IC95% 1,08;1,98). Quanto ao padrão saudável, identificou-se a frequência deste padrão foi maior em crianças com idade ≥ 24 meses (RP = 2,75; IC95% 1,83;4,14) e menor no sexo masculino (RP = 0,66; IC95% 0,49;0,87), e quando as mães tinham baixa escolaridade (RP = 0,61; IC95% 0,46;0,81).

**Tabela 3:** Análise ajustada entre os padrões alimentares não saudável e saudável de crianças residentes em favelas (n = 567), variáveis socioeconômicas da família e maternas relacionadas, Maceió, Alagoas, agosto de 2019-dezembro de 2021

**Variáveis**	**Padrão não saudável**						**Padrão saudável**					
	**Modelo 1**		**Modelo 2**		**Modelo 3**		**Modelo 1**		**Modelo 2**		**Modelo 3**	
	**RP** ^a^ **(IC**_95_ **%** ^b^ **)**	**p** **-valor**	**RP** ^a^ **(IC**_95_ **%** ^b^ **)**	**p-valor**	**RP** ^a^ **(IC**_95_ **%** ^b^ **)**	**p-valor**	**RP** ^a^ **(IC**_95_ **%** ^b^ **)**	**p-valor**	**RP** ^a^ **(IC**_95_ **%** ^b^ **)**	**p-valor**	**RP** ^a^ **(IC**_95_ **%** ^b^ **)**	**p-valor**
**Variáveis socioeconômicas da família**												
**Renda familiar** *per capita* ^c^ **(salários mínimos)**												
≥ 0,25	1,00											
< 0,25	1,26 (0,88;1,79)	0,202										
**Nível de pobreza** **(escore de Alvarez pontos)**												
Superior (45-54)							1,00					
Inferior e miséria (20-44)							0,76 (0,56;1,01)	0,063				
**Esgoto sanitário**												
Adequado	1,00											
Inadequado	1,16 (0,83;1,61)	0,380										
**Segurança alimentar**												
Segurança leve	1,00											
Insegurança moderada/grave	1,20 (0,85;1,69)	0,305										
**Variáveis maternas**												
**Idade (anos)**												
14-18									0,56 (0,24;1,29)	0,174		
19-29									1,00			
≥ 30									1,27 (0,95;1,69)	0,104		
**Escolaridade (anos)**												
> 9	1,00		1,00						1,00		1,00	
≤ 9	1,39 (1,01;1,93)	0,047	1,28 (0,93;1,76)	0,124					0,62 (0,46;0,82)	0,001	0,61 (0,46;0,81)	<0,001
**Qualidade de vida**												
**Comportamento psicológico**												
Adequado			1,00						1,00			
Inadequado			1,08 (0,79;1,45)	0,676					0,82 (0,59;1,13)	0,218		
**Senso de coerência**												
Forte			1,00						1,00			
Fraco			1,32 (0,96;1,82)	0,089					0,82 (0,65;1,16)	0,352		
**Excesso de peso**												
Não			1,00									
Sim			1,35 (0,85;2,14)	0,205								
**Obesidade abdominal**												
Não			1,00									
Sim			1,15 (0,70;1,87)	0,587								
**Variáveis relacionadas à criança**												
**Idade (meses)**												
< 24					1,00						1,00	
≥ 24					1,02 (1,01;1,03)	<0,001					2,75 (1,83;4,14)	<0,001
**Sexo**												
Feminino					1,00						1,00	
Masculino					1,46 (1,08;1,98)	0,015					0,66 (0,49;0,87)	0,004
**Peso ao nascer**												
Adequado					1,00						1,00	
Baixo peso					1,17 (0,70;1,94)	0,553					1,34 (0,89;2,01)	0,158
Elevado					1,23 (0,69;2,19)	0,478					0,46 (0,18;1,21)	0,116
**Histórico de amamentação**												
Sim					1,00						1,00	
Não					1,19 (0,78;1,77)	0,402					1,03 (0,74;1,44)	0,852
**Excesso de peso**												
Não											1,00	
Sim											0,70 (0,45;1,09)	0,112

a) RP: Razão de prevalências; b) IC_95_%: Intervalo de confiança de 95% da frequência relativa; c) Ponto de corte de 0,25 equivale a ¼ do salário mínimo.

## DISCUSSÃO

No presente estudo, identificaram-se dois padrões alimentares: saudável e não saudável. O maior consumo do padrão alimentar saudável associou-se à idade da criança ser ≥ 24 meses, enquanto o menor consumo, a crianças do sexo masculino e mães com menor escolaridade, resultados a reforçar a hipótese de baixo nível de escolaridade associado a escolhas alimentares menos saudáveis.^21^ O padrão não saudável teve seu menor consumo associado à idade da criança ≥ 24 meses e a crianças do sexo masculino. 

O número de padrões alimentares identificados assemelhou-se ao encontrado em um estudo que analisou o padrão alimentar de crianças brasileiras.^22^ O número de padrões possíveis de identificar em uma dada população é variável, depende da diversidade de grupos alimentares, do tamanho da amostra e das técnicas de extração dos padrões utilizadas nos estudos.^3^ Uma revisão sistemática, conduzida para identificar padrões alimentares em crianças de 7 a 10 anos e fatores associados, encontrou número de padrões alimentares variável, de dois a cinco, com predomínio de três.^3^

Em estudos de pesquisadores brasileiros^23^ e americanos,^24^ buscando relacionar padrões alimentares à síndrome metabólica e a problemas cardiovasculares, e identificar padrões alimentares derivados de análise *a posteriori,* como se decidiu na presente pesquisa, foram incluídos ovos nos padrões alimentares “não saudáveis”. A inclusão dessa fonte de proteínas de alto valor biológico nesse padrão, possivelmente, aconteceu pelo alto grau de vulnerabilidade social da população analisada, que utiliza embutidos e ovos como principais fontes proteicas em suas refeições, enquanto alternativas menos onerosas. A alta frequência de consumo de ovos pelas crianças influenciou as análises estatísticas, fazendo com que os ovos apresentassem maior correlação com alimentos não saudáveis.

Independentemente do número de padrões alimentares obtidos e alimentos componentes, cumpre identificar fatores associados a cada padrão. Estudo com crianças de 13 a 35 meses, em São Luís, Maranhão, concluiu que multiparidade, menor escolaridade materna e idade materna menor que 20 anos associaram-se ao menor consumo de alimentos saudáveis.^21^ Dados semelhantes encontram-se em pesquisa com 300 crianças de 4 a 24 meses, em Porto Alegre, Rio Grande do Sul, onde menor escolaridade materna associou-se a maior número de produtos ultraprocessados consumidos por crianças,^19^ corroborando esta casuística: menor escolaridade materna associou-se a redução do padrão alimentar saudável. Uma possível explicação para esse achado é o fato de mães com maior escolaridade apresentarem maior acesso às informações sobre práticas alimentares saudáveis. A escolaridade materna influencia o estilo de vida das crianças.^4^

Embora não tenham apresentado associação, sabe-se que fatores ambientais, nutricionais, psicológicos, sociais e culturais podem-se relacionar com comportamento alimentar.^25^ Crianças dependem dos pais/responsáveis para comprar/preparar suas refeições, seus hábitos alimentares são influenciados diretamente pelas crenças e cultura alimentar dos familiares.^25^ Nas famílias que não praticam uma dieta diversificada e se observa baixa frequência de consumo de alimentos saudáveis, há maiores chances de retardo no crescimento de suas crianças.^26^

Um dos achados da análise múltipla apontou associação da idade das crianças ≥ 24 meses com maior frequência de consumo de ambos os padrões, saudável e não saudável, semelhantemente a uma pesquisa realizada no Sul brasileiro, onde se observou associação entre padrões alimentares de crianças maiores que 12 meses;^27^ este achado baseou-se na maior independência dessas crianças para escolher alimentos e no acesso a maior variedade deles, quando comparadas às crianças de menor idade^28^ – embora, cumpre salientar, o público estudado foi de crianças de famílias em vulnerabilidade social, e principalmente, sem acesso a uma alimentação diversificada; ao alcançarem uma idade mais elevada, adquiririam aptidão fisiológica e autonomia nas escolhas alimentares, dentro das possibilidades existentes em seu ambiente.

Não se encontrar associação do sexo masculino com maior frequência de consumo do padrão alimentar não saudável no público infantil não é consenso na literatura. Estudos demonstraram que o sexo masculino se associa aos padrões saudável^29^ e não saudável;^1^ entretanto, pode-se hipotetizar que, em comunidades sob vulnerabilidade social, meninos apresentam maior independência e autonomia, inclusive para suas escolhas alimentares.

Quanto ao padrão saudável, filhos de mães com baixa escolaridade possuíam menor frequência de consumo dos alimentos desse padrão, fato também observado no estudo de coorte de São Luís, Maranhão.^21^ Baixa escolaridade dos pais pode indicar ausência de letramento nutricional adequado, que promova autocuidado satisfatório, em questões relacionadas à alimentação e nutrição das crianças.^19^

Segundo estudo realizado em Araraquara, São Paulo, no período 2015-2016, ao avaliar famílias beneficiárias ou não do Programa Bolsa Família, aquelas não contempladas pelo Programa estavam mais propensas a apresentar um padrão alimentar restrito, menos propensas a uma dieta saudável, independentemente da idade de seus membros.^30^ Pesquisa realizada no estado da Paraíba também verificou que crianças sob diferentes tipos de vulnerabilidade social possuíam maior probabilidade de apresentar um padrão alimentar não saudável.^5^

Esses dados reforçam o já esperado: entre crianças de famílias em vulnerabilidade social, menor escolaridade materna repercutiria negativamente em seu padrão alimentar. Hábitos alimentares adequados são extremamente importantes na infância porque, longitudinalmente, podem influenciar no estado nutricional e no desenvolvimento de doenças crônicas não transmissíveis.^5,29^

A formulação de políticas públicas voltadas à promoção da alimentação saudável para crianças em vulnerabilidade social, principalmente aquelas residentes em favelas, constitui desafio para governantes. Crucial ao desenvolvimento dessas políticas, a melhora na alimentação das crianças vivendo em ambientes desfavorecidos pode contribuir com a redução de desfechos desfavoráveis à saúde, como a obesidade.^4^

O estudo apresenta limitações: o uso de questionários extensos, cuja compreensão pode ser prejudicada pela baixa escolaridade materna; e o viés do entrevistador, conhecedor do público abordado, que pode influenciar no modo de as peguntas serem feitas, induzindo as respostas. Ambas as limitações implicam viés de informação, todavia minimizado ao se utilizar em questionários de instrumentos adotados para grandes inquéritos nacionais e aplicados por pesquisadores treinados. Outra limitação encontra-se no desenho transversal, que tanto impossibilita estabelecer relações de causalidade como propicia causalidade reversa, quando a associação entre as variáveis se apresenta de maneira diferente da expectável. O uso da análise múltipla atenuou esse viés. Utilizar um método retrospectivo na avaliação do consumo alimentar pode levar a erros nas respostas sobre alimentos consumidos, porque depende da memória do entrevistado. Contudo, trata-se de método amplamente utilizado em pesquisas populacionais^3,23^ para avaliação do consumo alimentar habitual de grupos, e a limitação do seu uso foi atenuada pela curta frequência adotada (última semana). Mais um entrave nos métodos do estudo, a lacuna temporal na coleta de dados não comprometeu a homogeneidade da amostra: indivíduos inseridos no estudo antes da pandemia não apresentaram diferenças estatisticamente significantes nas condições socioeconômicas e ambientais, comparados àqueles incluídos após o surto pandêmico (Tabela Suplementar 2).

Como ponto positivo desta pesquisa, encontra-se o cuidado metodológico na seleção dos participantes: foram recrutados todos os moradores das comunidades selecionadas e que atenderam aos critérios de elegibilidade, minimizando o risco do viés de seleção. Outro ponto forte foi o uso de instrumentos validados, na coleta de dados. Tais características demonstram a validade do estudo, interna e externa; seus resultados podem ser extrapolados para populações semelhantes no Brasil.

Conclui-se que a alimentação de crianças em vulnerabilidade social relacionou-se a fatores intrínsecos e extrínsecos à criança, a maior frequência do padrão saudável associou-se à idade ≥ 24 meses; e a menor frequência desse padrão, à baixa escolaridade materna e à criança do sexo masculino. O aumento da frequência do padrão não saudável foi prevalente em crianças ≥ 24 meses e do sexo masculino. Visando elucidar a causalidade das variáveis associadas à alimentação nessa população, fazem-se necessários estudos prospectivos.

## Tabela Suplementar 1

**Table:** Análise bruta entre o padrão alimentar não saudável e o padrão alimentar saudável de crianças residentes em favelas (n = 567), variáveis socioeconômicas da família e maternas relacionadas, Maceió, Alagoas, agosto de 2019-dezembro de 2021

Variáveis	Padrão não saudável(consumo > P75)			Padrão saudável(consumo > P75)		
	N (%)	RP^a^ bruta (IC_95_%^b^)	p-valor	N (%)	RP^a^ bruta (IC_95_%^b^)	p-valor
Variáveis socioeconômicas da família						
Renda familiar per capita^c^ ^c^(salários mínimos)						
≥ 0,25	36 (19,8)	1,00		44 (52,2)	1,00	
< 0,25	105 (27,3)	1,38 (0,99;1,93)	0,060	97 (25,2)	1,04 (0,76;1,42)	0,794
Nível de pobreza (escore de Alvarez pontos)						
Pobreza baixa superior (45 - 54)	36 (22,6)	1,00		48 (30,2)	1,00	
Pobreza baixa inferior e miséria (20 - 44)	105 (25,7)	1,14 (0,82;1,58)	0,449	93 (22,8)	0,76 (0,56;1,01)	0,063
Esgoto sanitário						
Adequado	59 (21,4)	1,00		73 (26,5)	1,00	
Inadequado	82 (28,2)	1,32 (0,98;1,76)	0,063	68 (23,4)	0,8 (0,66;1,18)	0,397
Segurança alimentar						
Segurança leve	45 (20,5)	1,00		60 (27,4)	1,00	
Insegurança moderada/grave	96 (27,6)	1,34 (0,98;1,83)	0,064	81 (23,3)	0,85(0,64;1,13)	0,267
Variáveis maternas						
Idade (anos)						
14 - 18	8 (19,5)	0,77 (0,40;1,48)	0,438	5 (12,2)	0,50 (0,21;1,17)	0,110
19 - 29	79 (25,2)	1,00		76 (24,3)	1,00	
≥ 30	54 (25,4)	1,00 (0,74;1,36)	0,977	60 (28,2)	1,16 (0,87;1,55)	0,316
Escolaridade (anos)						
> 9	44 (19,6)	1,00		74 (32,9)	1,00	
≤ 9	97 (28,4)	1,45 (1,06;1,99)	0,02	67 (19,6)	0,60 (0,45;0,79)	<0,001
Qualidade de vida						
Comportamento psicológico						
Adequado	83 (22,9)	1,00		98 (27,1)	1,00	
Inadequado	58 (28,3)	1,23 (0,92;165)	0,153	43 (21,0)	0,77(0,57;1,06)	0,113
Senso de coerência						
Forte	48 (20,4)	1,00		66 (28,1)	1,00	
Fraco	93 (28,0)	1,37 (1,01;1,86)	0,043	75 (22,6)	0,80 (0,60;1,07)	0,135
Excesso de peso						
Não	49 (20,1)	1,00		60 (24,6)	1,00	
Sim	92 (28,5)	1,42 (1,05;1,92)	0,024	81 (25,1)	1,02 (0,76;1,36)	0,894
Obesidade abdominal						
Não	37 (18,5)	1,00		51 (25,5)	1,00	
Sim	96 (27,3)	1,48 (1,05;2,07)	0,023	86 (24,5)	0,96(0,71;1,30)	0,794
Variáveis relacionadas à criança						
Idade (meses)						
< 24	18 (7,4)	1,00		31 (12,8)	1,00	
≥ 24	123 (38,0)	5,12 (3,21;8,17)	0,000	110 (33,9)	2,66 (1,85;3,82)	<0,001
Sexo						
Feminino	56 (20,3)	1,00		79 (28,6)	1,00	
Masculino	85 (29,2)	1,44 (1,07;1,93)	0,015	62 (21,3)	0,74 (0,56;0,99)	0,045
Peso ao nascer						
Adequado	104 (23,4)	1,00		110 (24,7)	1,00	
Baixo peso	12 (27,3)	1,17 (0,70;1,93)	0,554	17 (38,6)	1,56 (1,04;2,35)	0,031
Elevado	11 (34,4)	1,47 (0,89;2,44)	0,137	4 (12,5)	0,51 (0,20;1,28)	0,152
Excesso de peso						
Não	117 (26)	1,00		121 (26,1)	1,00	
Sim	24 (20,5)	0,79 (0,53;1,16)	0,233	20 (17,1)	0,64(0,41;0,97)	0,038
Histórico de amamentação						
Sim	38 (17,8)	1,00		42(19,7)	1,00	
Não	103 (29,1)	1,63 (1,17;2,27)	0,004	99 (28,0)	1,42 (1,03;1,95)	0,032

a) RP: Razão de prevalências; b) IC_95_%: Intervalo de confiança de 95%; c) Ponto de corte de 0,25 equivale a ¼ do salário mínimo.

## Tabela Suplementar 2

**Table:** Características socioeconômicas e ambientais das famílias na coleta de dados no período pré-pandemia e após o surto pandêmico

Variáveis	Total		Pré-pandemia		Após surto pandêmico		p-valor
	N = 567	%	N = 354	%	N = 213	%	
Renda familiar per capita^a^ (salários mínimos)							
≥ 0,25	172	30,3	107	30,3	64	30,1	0,518
< 0,25	395	69,7	247	69,7	149	69,9	
Nível de pobreza (escore de Alvarez pontos)							
Superior (45 - 54)	152	26,8	103	29,0	60	28,0	0,313
Inferior e miséria (20 - 44)	415	73,2	251	71,0	153	72,0	
Água de consumo							
Adequada	174	30,7	110	31,0	65	30,5	0,893
Inadequado	393	69,3	244	69,0	148	69,5	
Esgoto sanitário							
Adequado	183	32,3	110	31,1	66	31,0	0,985
Inadequada	384	67,7	244	68,9	147	69,0	
Manejo do lixo							
Adequado	193	34,0	121	34,2	71	33,5	0,644
Inadequado	374	66,0	233	65,8	142	66,5	
Segurança alimentar e nutricional							
Segurança leve	218	38,4	134	38,0	83	39,0	0,658
Insegurança moderada/grave	349	61,6	220	62,0	130	61,0	

a) Ponto de corte de 0,25 equivale a ¼ do salário mínimo.
